# Segmental Upregulation of ASIC1 Channels in the Formalin Acute Pain Mouse Model

**DOI:** 10.3390/ph15121539

**Published:** 2022-12-12

**Authors:** María Natalia Gobetto, Libia Catalina Salinas Castellanos, Natalia Estefanía Contreras, Alejandro Omar Sodero, Damián Alejandro Cambiagno, Georgina Oriana Mingolo Malnati, Mayra Micaela Montes, Osvaldo Daniel Uchitel, Carina Weissmann

**Affiliations:** 1 Instituto de Fisiología Biologia Moleculary Neurociencias-IFIBYNE-UBA-CONICET, LFBM, Bueno Aires CP1428, Argentina; 2 Institute of Biomedical Research (BIOMED), Pontifical Catholic University of Argentina (UCA) and National Scientific and Technical Research Council (CONICET), Buenos Aires C1107AFF, Argentina; 3 Unidad de Estudios Agropecuarios (UDEA), INTA-CONICET, Córdoba X5020ICA, Argentina

**Keywords:** formalin mouse model, ASIC1 channels, miR485-5p, miRNA, pain

## Abstract

Background: Hindpaw injection of formalin in rodents is used to assess acute persistent pain. The response to formalin is biphasic. The initial response (first minutes) is thought to be linked to inflammatory, peripheral mechanisms, while the latter (around 30 min after the injection), is linked to central mechanisms. This model is useful to analyze the effect of drugs at one or both phases, and the involvement of ion channels in the response. Acid-sensing ion channels (ASICs) regulate synaptic activities and play important roles in pain conditions. Recently, psalmotoxin-1 (Pctx-1), a toxin that inhibits ASIC1a-constituted channels, and antisense ASIC1a-RNA, intrathecal administered in mice were shown to affect both phases of the test. Methods: The mouse formalin test was performed on C57/BL6 7- to 9-week-old mice. Behavioral tests were conducted and tissue was extracted to detect proteins (ASIC1 and pERK) and ASIC1-mRNA and mir485-5p levels. Results: The injection of formalin was accompanied by an increase in ASIC1 levels. This was detected at the contralateral anterior cingulate cortex (ACC) compared to the ipsilateral side, and both sides of the ACC of vehicle-injected animals. At the spinal cord and dorsal root ganglia, ASIC1 levels followed a gradient stronger at lumbar (L) 3 and decreased towards L5. Gender differences were detected at the ACC; with female mice showing higher ASIC1a levels at the ACC. No significant changes in ASIC1-mRNA levels were detected. Evidence suggests ASIC1 upregulation depends on regulatory microRNAs. Conclusion: This work highlights the important role of ASIC1 in pain and the potential role of pharmacological therapies aimed at this channel.

## 1. Introduction

ASICs (Acid Sensing Ion Channels) are involved in fear behavior, learning and memory functions, as well as pain sensation [1], and synaptic transmission in general [2]. ASIC channels are part of the amiloride-sensitive epithelial sodium channel (ENaC)/degenerin (DEG) superfamily of ion channels [1]. In rodents and humans, five genes, including ASIC 1–5 (Accn1–5) encode at least seven ASICs subtypes, ASICs (1a, 1b, 2a, 2b, 3, 4 and 5) [2]. ASICs are trimers, with both homo and heterotrimers as feasible arrangements [3]. The sensitivity to external protons, the activation and inactivation kinetics, and the pharmacology vary according to the ASIC subtypes and the subunit composition of the channel complex, with pH 0.5 for activation ranging from 4.0 to 6.8 and activation thresholds as high as pH 7.2 [4]. While most ASICs are impermeable to Ca^2+^, the ASIC1a subunit forms permeable heteromeric channels with ASIC2b, and also Ca^2+^ -permeable homomeric channels. Homomeric ASIC1a, and heteromeric ASIC1a/ASIC2a or ASIC1a/ASIC2b show widespread expression throughout the central and peripheral nervous systems (PNS), whereas ASIC1b and ASIC3 are largely restricted to the peripheral nervous system [2]. ASICs are also expressed in pain-processing areas of the central nervous system (CNS). Administration of an ASIC1a-specific toxin, PcTx-1 or Mambalgin-1 to the CNS inhibited pain behavior, indicating a role of central ASIC1a in pain sensation, in addition to the role of ASICs as pH sensors in the PNS [1].

Many studies have tried to elucidate the role of the different ASIC isoforms using different pain models as well as ASIC knock-out animals. Results from these experiments are far from conclusive. Analyzing the role of the different isoforms in pain is complex: the different isoforms are distributed differently in the various species analyzed; the isoforms are involved in distinct modes of sensation; and according to the tools used, function, distribution, protein, and mRNA levels have not always been analyzed comprehensively. Upregulation of ASIC channels has been described in the literature. In some studies this is described through functional experiments, without determination of the increase in protein levels [5]; likewise, changes in RNA levels have been analyzed without a reference to the protein content [6]. Moreover, most work published has used an anti-ASIC1 antibody that cannot distinguish between ASIC1a or ASIC1b isoforms. To the best of our knowledge, there is only one ASIC1a specific conformational antibody generated recently with high specificity for the human ASIC1a isoform [7] which has been used in few publications so far.

Identifying the specific isoforms involved in a particular pain modality is key to develop specific pharmacological tools and provide proper therapies. Additionally, the role of micro RNAs in modulating proteins related to pain development is now well recognized [8,9,10], and recently, the mechanism in which this regulation can occur was described for ASIC1 [11].

In this study, we analyzed the formalin acute mouse pain model to assess the contribution of ASIC1 channels, and the mRNA levels of the isoform involved in the pain pathway following the analgesia shown for the treatment of animals with an ASIC1a inhibitor. The model has been previously used to assess the contribution in the PNS or CNS of different channels to pain [12,13,14].

## 2. Results

### 2.1. Formalin Test to Assess the Pain Pathway in Mice

The formalin test in mice has been described to trigger a biphasic response in the first minutes and a later response around 30 min after the injection. The formalin model has been used to test different compounds affecting either one or both phases to analyze the underlying mechanisms governing acute/inflammatory or persistent pain.

**Figure 1:**
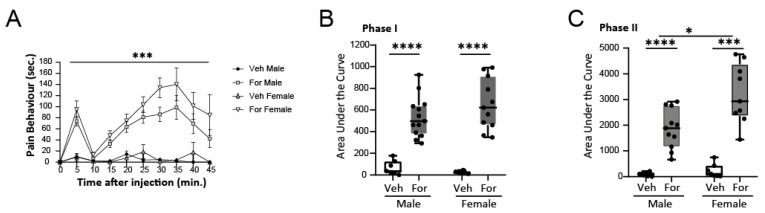
Response to formalin test in 8-week-old mice. (**A**) Pain behavior (in seconds) at different time points after formalin (For) male (white squares) and female (triangle facing down), or Veh male (black squares) and female (triangle facing up) -injected mice). ANOVA repeated measures, *** *p* < 0.001. Area under the curve for Phase I (**B**) from 0 to 15 min or Phase II (**C**) 15 to 45 min of the For or Veh test described in (**A**) in females and males (**C**). Two-way ANOVA, **** *p* < 0.0001; * *p* < 0.05.

We used this test in 8-week-old male and female C57BL/6 mice. As shown in Figure 1, the response measured, as described in the literature, was biphasic showing significant differences between male and female mice for the second phase, associated mostly with central sensitization.

### 2.2. Elevated ASIC1 Protein in Central and Peripheral Areas in Pain

Among drugs that modulate one or both phases, amiloride, a non-specific ASIC inhibitor, was documented to display analgesic effects in the formalin model [15,16]. Furthermore, another ASIC channel modulator, Psalmotoxin-1 (Pctx-1), specific for ASIC1a-constituted channels has been reported to affect both phases of the formalin test [17]. In addition, ASIC1a antisense injected into animals before the formalin test has also been shown to affect both phases [17]. However, ASIC1 protein levels have not been analyzed in all the studies, and ASIC1 RNA levels have given conflicting results [16]. We analyzed ASIC1 protein levels in control and formalin injected mice at the peripheral dorsal root ganglia (DRGs), central spinal cord (SC), and supraspinal anterior cingulate cortex (ACC). In addition, we decided to segment the ACC in ipsilateral (Ip) or contralateral (Con) areas to the injection site. Segments in the lumbar (L) area of the SC and DRGs (L3, L4, L5, Ip and Con) were analyzed independently to avoid a dilution effect, and target the regions that contribute to the mouse sciatic nerve.

**Figure 2:**
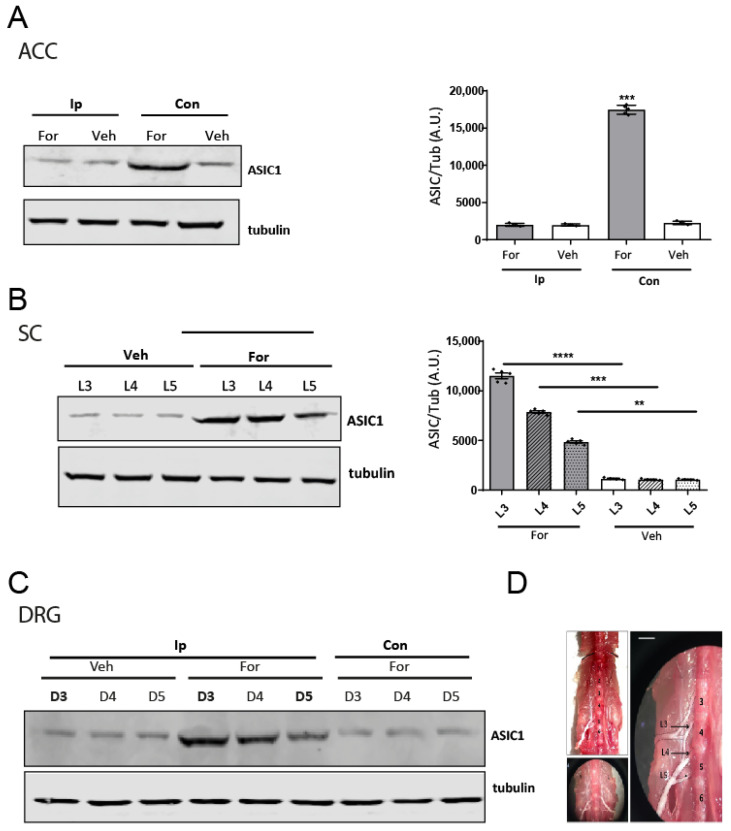
ASIC1 levels in the pain pathway. (**A**) Representative membrane of lysates of ACC tissue from formalin (For)- or vehicle (Veh)- injected male mice at Ip (ipsilateral) or Con (contralateral) sides to the injection detected using ASIC1 and tubulin antibodies (left), and plot of the results obtained from membranes for ASIC1/tubulin detected levels (right) (4–5 animals used per condition). (**B**) Representative membrane of lysates of SC tissue from For- or Veh- injected mice at different lumbar (3, 4, 5) levels detected using ASIC1 and tubulin antibodies (left), and plot of the results obtained from membranes for ASIC1/tubulin detected levels (right) (4–5 animals used per condition). Notice the gradient decrease from level 3 to 5. (**C**) Representative membrane of lysates of a pool of DRG tissue from different lumbar (3, 4, 5) levels in For- or Veh- injected male mice at Ip or Con sides to the injection detected using ASIC1 and tubulin antibodies. Notice the same gradient pattern as in (**B**,**D**) Images of the regions dissected (SC, DRG segments) used for the experiments in (**B**,**C**). One-way ANOVA, **** *p* < 0.0001; *** *p* < 0.001 ** *p* < 0.01. Scale bar 5 mm.

As shown in Figure 2, we detected differences in ASIC1 levels at the ACC contralateral to the formalin injection compared to the ipsilateral, or contralateral veh-injected mice (Figure 2A,D). In the case of the SC, we could detect differences in ASIC1 levels following a gradient decreasing from L3 to L5 segments (Figure 2B,D), a pattern that was also encountered in ipsilateral DRGs (Figure 2C,D).

Female tissues processed after the test showed the same increases for ASIC1 subunits. Moreover, ASIC1 levels at the contralateral ACC of formalin-injected females showed significant increases compared to males (Figure S2A). This was not the case at the L3 of SC or DRGs (Figure S2B,C).

### 2.3. Signaling Down-Stream ASIC1a

**Figure 3:**
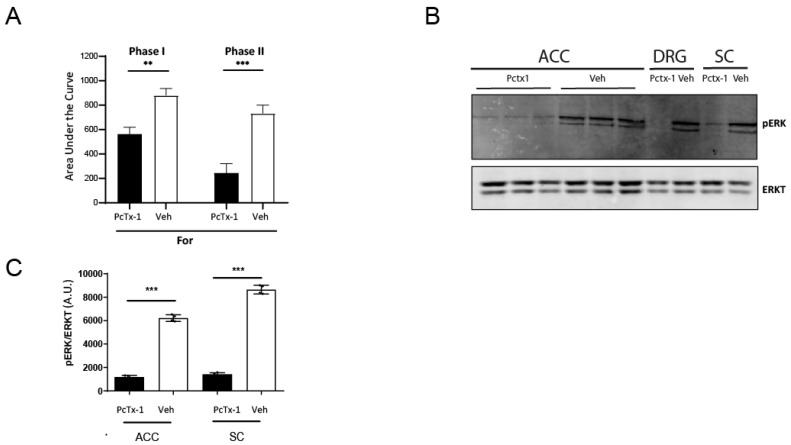
Effect of an intrathecal injection of Pctx-1 on the formalin test. (**A**) Area under the curve for the early (from 0 to 15 min) or late (15 to 45 min) phase of the formalin test in male mice injected with Pctx-1 (0.1 nanomols, grey) or vehicle (black) previous to the injection of formalin (two-way ANOVA, *** *p* < 0.001; ** *p* < 0.01 (**B**) Representative membrane of lysates of ACC, DRG (pools) and SC tissue from the animals tested in A detected with pERK antibody (upper panel) and normalized to total ERK levels (lower panel), and (**C**) plot of the results obtained from membranes for pERK/tubulin detected levels. One-way ANOVA, *** *p* < 0.001; *n* = 4.

We previously documented the contribution of ASIC1a channels to the activation of the ERK pathway [18,19]. This pathway is recognized as one involved in pain [20,21,22,23,24,25]. To evaluate whether ERK pathway modulates the formalin response, we assayed the early and late phase from animals pre-treated with an intrathecal injection of the ASIC1a inhibitor peptide, Pctx-1, or vehicle before the formalin test. As shown in Figure 3, an intrathecal injection of the peptide was able to decrease the response to formalin in the early and late phases (Figure 3A), similar to the results reported by Mazzuca et al. [17]. Moreover, ERK phosphorylation decreased in Pctx-1-pretreated animals in all the areas analyzed (ACC, SC, DRGs; Figure 3B), significantly (Figure 3C), consistent with an increase in phosphorylated ERK in For-injected vs veh-injected animals as described by Okine et al. using the rat formalin model [26] and in our experiments (Figure S1). This result further supports the involvement of the ASIC1a channel in the pain pathway and provides target molecules operating downstream, as well as the possibility of regulating the pathway with channel inhibitors.

### 2.4. Regulation of ASIC1 Protein: Potential Upstream Mechanisms Involved

**Figure 4:**
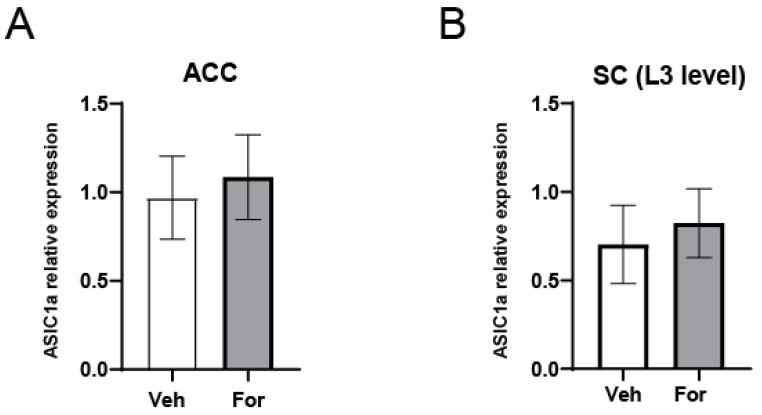
ASIC1a mRNA levels in the pain pathway. (**A**) Plots of ASIC1a mRNA levels in ACC contralateral regions injected with formalin or vehicle (PBS). (**B**) Plot of ASIC1a mRNA levels in the L3 segment of the SC in formalin or PBS injected animals. Two-tailed unpaired t-test (experiments in technical triplicates; four animals used per condition).

To further analyze the mechanism responsible for the increase in ASIC1 protein levels, we analyzed whether it correlated with an increase in ASIC1 mRNA levels. In contrast to our expectation, mRNA levels for ASIC1a in ACC and SC tissues were unchanged in For- or Veh-injected male mice (Figure 4A). DRGs showed the same tendency (not shown). Thus, no increase in mRNA was detected correlating with the protein increase. ASIC1b RNA was barely detectable at the ACC or SC consistent with the isoform distribution in literature, and did not change after formalin injection.

Recently, Xu et al. documented the upregulation of ASIC1 channels triggered by a decrease in the non-coding miR-485-5p RNA in an inflammatory pain model [11]. Therefore, we tested whether this mechanism could be responsible for the differences detected at the protein level in the formalin model.

**Figure 5:**
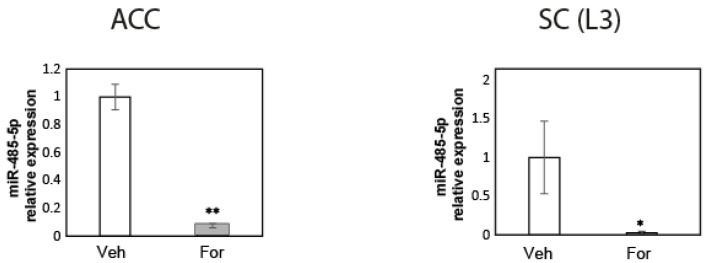
miR-485-5p levels in the pain pathway. Representative plot of miR-485-5p relativized to GAPDH (2ddCT) in ACC contralateral regions (**left**), and SC at L3 (**right**). The plots represent one of five (ACC) or three (SC) biological replicates showing the same differences between Veh- and For- treated samples. One of three biological replicates ± SE is shown. * *p* < 0.05, ** *p* < 0.01, two-tailed unpaired *t*-test.

As shown in Figure 5, a decrease in miR-485-5p was detected in tissues obtained from the formalin-injected animals. Moreover, preliminary results from DRG pools showed the same trend (Figure S1B). These results suggest that the decrease in miR485-5p levels might be involved in the upregulation of ASIC1 channels in this pain model in male mice by inhibiting the translation of its mRNA, similar to what was described by Xu et al.

Since ASIC1 levels at the ACC were higher in female than male mice, we tested whether miR-485-5p might differ. We detected higher levels of miR-485-5p in non-treated males compared to females (Figure S2D), suggesting that its basal repression in female mice could be in a miR-485-5p-independent manner. Therefore, a different mechanism might be responsible for the regulation of ASIC1 channels in female mice.

**Figure 6:**
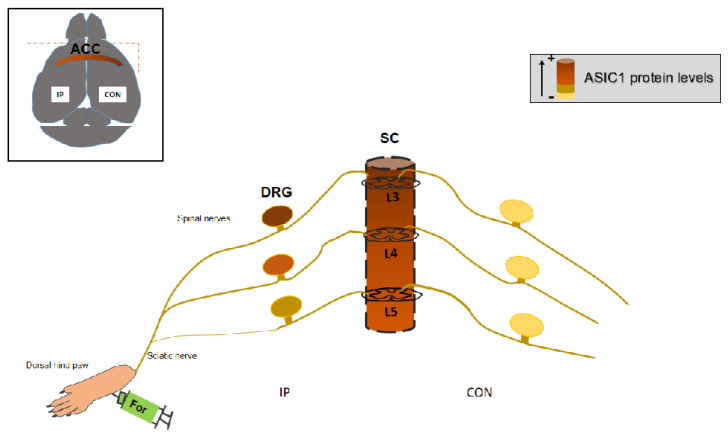
Schematic representation of ASIC1 upregulation in ACC, SC and DRG in the formalin mouse model of pain. At the cortex, the ACC contralateral to the injection (left hind paw) shows a higher ASIC1 protein level. At the SC and DRGs, there is a gradient decreasing from L3 to L5 lumbar segments. Spinal nerves contribution to the sciatic nerve is represented with lines, thicker for those contributing to a greater extent. ASIC1 protein levels are represented showing an increase with more intense color. (IP, ipsilateral; CON, contralateral; ACC, Anterior Cingulate Cortex; DRG (Dorsal Root Ganglia; SC, Spinal Cord; L3, 4, 5, lumbar 3, 4, 5).

A schematic representation of the difference in ASIC1 levels in the formalin-pain model is shown in Figure 6.

## 3. Discussion

Animal models of pain have aided in the analysis of the involvement of ion channels as potential targets for pain therapies [27,28]. Among these, ASIC1 channels have been documented to play a role in several models of pain. In the complete Freund’s adjuvant (CFA) injection model, spinal infusion with specific inhibitors or antisense oligonucleotides to ASIC1a markedly attenuated the CFA-induced thermal and mechanical hypersensitivity, linking ASIC1a channels to inflammatory pain hypersensitivity [5]. Although ASIC1a upregulation was found at the SDH level, and not in DRGs, the authors discussed the fact that upregulation in DRGs could have occurred at the peripheral terminals or cell bodies, as DRG terminals in the spinal cord only were analyzed [5]. In fact, ASIC1a expression in DRG neurons is upregulated after peripheral inflammation [29]. Using another animal model, that of amputation (of the digit and hind paw), as well as the formalin model, Wei et al. revealed a key role for ERK-dependent neuronal plasticity within the ACC in the development of neuropathic pain [24]. Okine et al. documented the activation of ERK at the ACC in the formalin rat model connected to the G-protein coupled receptor, GPR5, with endogenous activation of GPR55 signaling and increased ERK phosphorylation in the ACC facilitating inflammatory pain via top-down modulation of descending pain control [26]. The phosphorylation of the ERK kinase has also been linked to the activation of ASIC1 channels [19,30]. We showed that ASIC1 channels contribute to the ERK signaling pathway in this pain model (Figure 3B and Figure S1) which further links the channel to the pain pathway.

Many examples in the literature contrast the fact that ASIC1a knock-out mice are involved in pain as that induced by carrageenan in a mouse model, whereas ASIC1a RNA levels show no changes whatsoever [31]. Moreover, in the nerve ligation model, even though Pctx-1 showed an analgesic effect [17], ASIC1a RNA levels showed no increase as measured by Jeong et al. [16]. Staniland et al., analyzed ASIC1, ASIC2, and ASIC3 knock-out animals, and documented ASIC1a knock-out animals increased the spontaneous pain activity in the second phase of the formalin test [32], concluding that ASIC1 is involved in modulating the response evoked by formalin. Furthermore, Mazzuca et al. showed the analgesic effects of Pctx-1 on both phases of the formalin test, connecting the role of ASIC1a channels to the encephalin system [17]. In addition, other channels have been shown to contribute to pain induced by formalin in mice and in rats. Martinez-Rojas et al. showed that TRPA1 in rats DRG and spinal cord plays a relevant role in formalin-induced long-lasting secondary nociceptive hypersensitivity. Moreover, TRPA1 blockers administered locally or centrally, before or after formalin treatment, were shown to alter (reverse or decrease) the effects of formalin treatment [13]. Li-Juan et al. analyzed the involvement of TRPV1 channels in the model using dissociated DRGs. They showed that TRPV1 receptors are likely to be in volved in activation of a subpopulation of primary nociceptors by formalin and contribute mainly to phase 2 of formalin nociception [33]. Further, experiments by Kanai et al. showed the spinal contribution of TRPV1 channels contributed to both phases when a specific TRPV1 blocker was administered intrathecally [14]. Non-steroidal anti-inflammatory drugs were also tested in the model. Malmberg et al. showed a decrease in the second formalin phase in rats after intrathecal administration of NSAIDs [34]. Interestingly, NSAIDs have been documented to modulate ASIC channels [35].

We documented the upregulation of ASIC1 protein in the different tissues analyzed in the formalin model (Figure 2). In addition, we showed that the upregulation was segmental producing a gradient pattern of ASIC1 protein. The segments used at the lumbar area are those from spinal nerves that contribute most to the sciatic nerves in mice, which differ from rats [36]. As analyzed by Rigaud et al., in mice, the L5 spinal nerve appears to make a large contribution, but in fact, this is not the case: there is a typically slight contribution from the L5 spinal nerve to the sciatic nerve, and strain-dependent variability in segmental contributes to the sciatic nerve and may account in part for genetic differences in pain, as in the case for nerve ligation experiments [36]. Therefore, we focused on L3, L4 and L5 segments separately. Indeed, we found that spinal nerves, as well as SC segments, contributing most to the sciatic nerve, -afferent pathway from the hind paw-, showed the highest differences in ASIC1a levels. This effect would, thus, reside in different segments that contribute differently to the sciatic nerve conformation, according to the species, and strains. In turn, this fact should be considered in studies, as the effect could dilute by analyzing all tissues combined. Altogether it shows how a noxious stimulus is conveyed, as well as the most affected area that might be treated. Such segmental gradients in DRGs have been described before for other molecules. A transcription factor in different DRG varied according to the segments analyzed in a mouse model of nerve injury [37]. Papalampropoulou-Tsiridou et al. showed that the pattern of ASIC mRNA isoforms and their accumulation varied among DRG depending on their segmental level, and interpreted this result as a differential regulatory mechanism between afferent types and anatomical location [6]. We showed that ASIC1a mRNA levels remained unchanged in this model (Figure 4). The mechanism responsible for ASIC1 protein upregulation has been characterized recently in a rat pain model, showing a role for micro RNAs. Regulation of protein expression by miRNAs has been the focus of various studies in the last years. The study of, at least some, miRNAs expression and degradation show fast kinetics that in turn reflect on fast changes in protein expression levels [38,39]. miRNAs have also received attention in the field of pain lately as key molecules responsible for changes in gene transcripts associated with pain. In fact, the formalin model has been used to investigate miRNA changes. miRNA-124a has been described as being significantly downregulated in the formalin model already 1 h after injection in central SC tissue [40]. miR-485-5p was reported as downregulated in DRGs in the CFA-inflammation mouse pain model at 8 h and at later time points after the stimulus. This downregulation was shown to be responsible for an increase in ASIC1 protein levels; we showed the same miRNA might also be responsible for the mechanism in the formalin model (Figure 5). The study did not mention earlier time points or ASIC1 mRNA levels. Although beyond the scope of this study, the mechanism by which microRNAs are regulated could relate to the increase in cytokine levels in inflammatory conditions [41,42,43]. Fast changes in protein synthesis in models of pain have been documented before. In fact, the cytokine IL-6 and NGF were shown to induce fast changes in protein levels through the eukaryotic initiation factor (EIF) EIF4f complex, signaling via ERK kinase and mTOR respectively [44]. Thus, the use of rapamycin to inhibit protein translation has been linked to pain relief, and detected in DRGs and spinal cord [44] as well as in the ACC [45].

Moreover, ERK phosphorylation was detected after 15 min treatment of DRG cultures [44] and was linked to the MEK1 pathway. The persistence of these changes has been suggested to underlie the change towards chronic states. It remains to be elucidated whether ASIC1a levels change back to initial levels at extended time points after FOR assays, and if these could relate to chronic models as evaluated by Mazzuca et al. (Mazzuca et al., 2007).

Another aspect analyzed preliminary in this work was that of differences in pain thresholds in male and female mice. We have observed higher responses to pain in the second phase of the formalin test for female versus male mice (Figure 1), and higher ASIC1 levels at the ACC for female mice (Figure S2). Sex differences in response to pain have been documented in the formalin model [46,47,48]. Moreover, the effect of amiloride in the formalin model analyzed [15] showed differences between genders [49]. Thus, special mechanisms governing central sensitization are involved in pain and in gender differences in pain (second phase). In agreement, Kim et al. identified a later phase of the formalin test which was greater in female mice than in male mice regardless of the stage of the estrus cycle [47]. Gaumond et al. documented a role for sex hormones in the perception of pain analyzing differences between nociceptive responses of castrated males and ovariectomized females [46]. In addition, miRNAs may be differentially accumulated between males and females in physiological [50,51] and pathological conditions [52]. Future studies will further determine putative different mechanisms of regulation of ASIC1 by miRNA485-5p among them.

Different NS structures compose the pain pathway and play a role in pain perceptions. We detected an upregulation of ASIC1a channels in peripheral and central structures. We showed that intrathecal administration of Pctx-1 modulated this effect. Mazzucca et al. had analyzed the different modes of administration of drugs to decrease ASIC1a levels showing that Pctx-1, a peptide, was only effective through intrathecal or intracerebroventricular administration, but not via subcutaneous or intraperitoneal injection [17]. Intrathecal administration of anti-ASIC1a mRNA showed similar results [17]. Whether other channels play a greater contribution in the PNS, as ASIC3, TRPV1 and others are highly represented in DRGs, or if the upregulated ASIC1a subunit is not properly delivered to the plasma membrane, or forms ASIC channels in combination with other subunits that would render the channel insensible to Pctx-1, remains to be investigated. Nonetheless, Voilley et al. analyzed the use of NSAIDs in the model of Complete Freund adjuvant-administration to induce inflammation and pain in mice, and found that injection of ibuprofen and flurbiprofen inhibited ASIC1a activation and the upregulation of the channel [35].

It has been proposed that neuropathic pain, as from an injury, leads to facilitation mechanisms in the brain that ultimately sensitize pain transmission neurons in the spinal cord; if CNS facilitation persists even after the healing of the injury, it contributes to drive the neural and behavioral manifestations of chronic pain [53]. The induction of central sensitization has been identified at the spinal cord level (dorsal horn neurons, second-order neurons) and in brain regions like the anterior cingulate cortex (ACC), prefrontal areas, thalamus and parietal association area. The ACC is thought to be important for acute pain perception as well as the development of chronic pain after peripheral nerve injury. Activation of c-fos in the contralateral ACC has been documented in a model of peripheral nerve injury [54]. In our experimental study (see Figure 2 and Figure S1) we detected an increase in ASIC1 protein, together with a decrease in miR485-5p levels, and an increase in pERK levels in the ACC contralateral to the formalin injection. Nevertheless, bilateral activation of ACC neurons has also been documented upon noxious stimulation of either hind paw [55]; and bilateral activation of ERK was described by Cao et al. using the formalin model, in rat, with a higher formalin concentration, and analyzed at different time points after the formalin injection than the ones used in this work [25]. Whether the different noxious stimulus, and concentrations (2.5% versus 5% formalin), or molecule-like channels are affected differently remains to be further analyzed. Changes in the effects triggered by different concentrations of formalin have been described [56,57]. In addition, the corpus callosum could play a bigger role in some instances to explain the detection of some effects on both sides [58].

All in all, we propose a model in which the differences in ASIC1 protein levels in different areas of the CNS and PNS are related to pain in the acute mouse pain model (Figure 6, scheme). Finally, a correlation with the downregulation of miR-485-5p may mechanistically explain ASIC1 protein levels in male mice. Future studies will determine how this might be modulated to target different pain conditions.

## 4. Materials and Methods

### 4.1. Animal Model and Formalin Test

Animals were kept in the animal facility of the Faculty of Exact and Natural Sciences (University of Buenos Aires, Buenos Aires, Argentina). All experiments involving mice were performed according to national guidelines and were approved by local ethics committees (CICUAL). We used male and female C57/BL6 mice, aged 7 to 9 weeks (20–30 g) (*n* = 9 to 13 mice randomly divided into groups depending on the experimental design). The mice were housed in groups of 4 per cage on a 12 h:12 h light–dark cycle at a constant room temperature of 22 ± 1 °C and in conditions of relative humidity of 65% and provided with food and water ad libitum. Previous to any animal testing, we confirmed that mice presented a normal behavior, using supervised protocols (codes 112, 131), approved by the CICUAL Experimentation Ethical Committee, indicating that mice had a healthy status. The mice received a subcutaneous injection of formalin (20 µL at 2.5% concentration—MERCK) or vehicle (PBS) into the left hind paw using a syringe with a 30-Gauge needle. Pain behavior was scored using a stopwatch when flinching, licking and flinching continued by licking or shaking of the paw were initiated. The timer was stopped once pain behavior ceased. Before the injection, mice were acclimatized for 1 h in the same 16 × 16 cm glass chambers used to detect the behavior response. The behavior response to the formalin test was performed during the daytime only (13:00−18:00 h). The first (early) phase of the test was defined as the time between zero and 15 min after the injection; the second (late) phase: between 15 and 45 min. The area under the curve (AUC) was calculated in relation to the pain response (sec) over time [40,59].

Another group of male animals received an intrathecal injection of 10 μL of Psalmotoxin-1 0.1 nmol (PcTx-1, Alomone Labs) using a Hamilton syringe with a 30-Gauge needle. PcTx-1 was dissolved on the day of experimentation in a physiological saline solution (mM: NaCl 140, KCl 5, MgCl_2_ 2, CaCl_2_ 2, HEPES 10 pH 7.35). BSA (1 mg/mL) was added to the solution of PcTx-1 to prevent non-specific adsorption of the toxin. Vehicle-treated animals, in this case, received only the physiological saline solution. The injections were performed under light isoflurane anesthesia between spinal segments L5 and L6 according to the method described by Hylden and Wilcox for mice [60]. All experimental results were collected and registered by an experimenter blinded to the treatment.

### 4.2. Tissue Collection

Mice were sacrificed in deep avertin anesthesia (2% Tribromoethanol, SIGMA) at the end of the behavioral assessment (45 min after FOR or VEH injection). ACC (ipsilateral (Ip) and contralateral (Con) to the injection site; SC lumbar (L) segments L3, L4, and L5 and DRGs (Ip and Con, as well as D3, 4, and 5) were dissected and separated for western blot analysis. The tissue was shock-frozen in liquid nitrogen for storage at −80 °C before further processing. For ACC and SC, an *n* = 4/5 per group was used to obtain statistical differences. In the case of DRGs, a pool of at least 3 DRGs corresponding to the same segment was used per experiment to detect a signal, for this reason, representative results only are shown.

### 4.3. Protein Analysis

For protein analysis, tissues were processed using a manual homogenizer with protease inhibitors. The suspension was centrifuged for 10 min at 10,000 rpm and the supernatant was used with loading buffer 6x in the electrophoresis run as described before (Salinas et al., 2020). The following primary antibodies were used: rabbit polyclonal anti ASIC1 (Alomone ASC-014, 1:1000); mouse monoclonal anti-tubulin (DM1a; Cell signaling #3873, 1:5000); rabbit polyclonal anti-total ERK (Santa Cruz, C9, 1:500); rabbit polyclonal anti phosphoERK (Cell Signaling, SC-7383, 1:500). Reactive bands were detected by the LI-COR Odyssey system, using secondary antibodies: 926-68073 IRDye 680RD Donkey anti-Rabbit IgG or 926-32212 IRDye 800CW Donkey anti-Mouse. Images were taken using the LI-COR Odyssey system and quantified with ImageJ software (NIH, Bethesda, MD, USA).

### 4.4. Quantitative PCR

Ribonucleic acid (RNA) samples were prepared from tissue using Trizol reagent (Thermo Fisher, Waltham, MA, USA) according to manufacturer guidelines. Then, 200 ng of total RNA was subjected to reverse transcription using the iScript™ cDNA Synthesis Kit—Bio-Rad to obtain 10 ng/mL cDNA. Next, the real-time polymerase chain reaction (PCR) analysis was performed based on the SYBR Green PCR Master Mix from the Biorad assay with the StepOne Real-Time PCR System (Life Technologies, Carlsbad, CA, USA). The ASIC1a and ASIC1b primers used were those used by Papalampropoulou-Tsiridou et al. [6]. Primers to amplify miR-485-5p were those used by Gao et al. (Gao et al., 2019). The expression of the gene of interest was normalized using GAPDH as a housekeeping gene as used in [61], to avoid differences due to possible RNA degradation or different reverse transcription efficacy, the relative expression levels were calculated using the Ct method.

### 4.5. Data Analysis and Figure Preparation

Data were analyzed by Student’s *t*-test, ANOVA, and Dunnet post hoc tests and plotted using Graph Pad Prism version 8 (Graph Pad Software Inc., La Jolla, San Jose, CA, USA). Images were designed using Adobe Illustrator CC version 10 software. In the case of miRNA assays, one representative assay (of three biological replicates) from all the assays performed is given and the numbers that correspond to each group is indicated in the legend for the biological samples tested.

## Data Availability

Data is contained within the article and supplementary material.

## Supplementary Materials

The following supporting information can be downloaded at: https://www.mdpi.com/article/10.3390/ph15121539/s1, Figure S1: ASIC1 and pERK levels; Figure S2: ASIC1 and miR-485-5p levels in male and female mice.
