# Supplementary material for: Segmental Upregulation of ASIC1 Channels in the Formalin Acute Pain Mouse Model
Source: Pharmaceuticals (Basel). 2022 Dec 12;15(12):1539. doi: 10.3390/ph15121539 (PMC9784454; doi:10.3390/ph15121539)
Supplement: Supplementary file 1 [file pharmaceuticals-15-01539-s001.zip › pharmaceuticals-2020119-supplementary.pdf]

Supplementary Figures

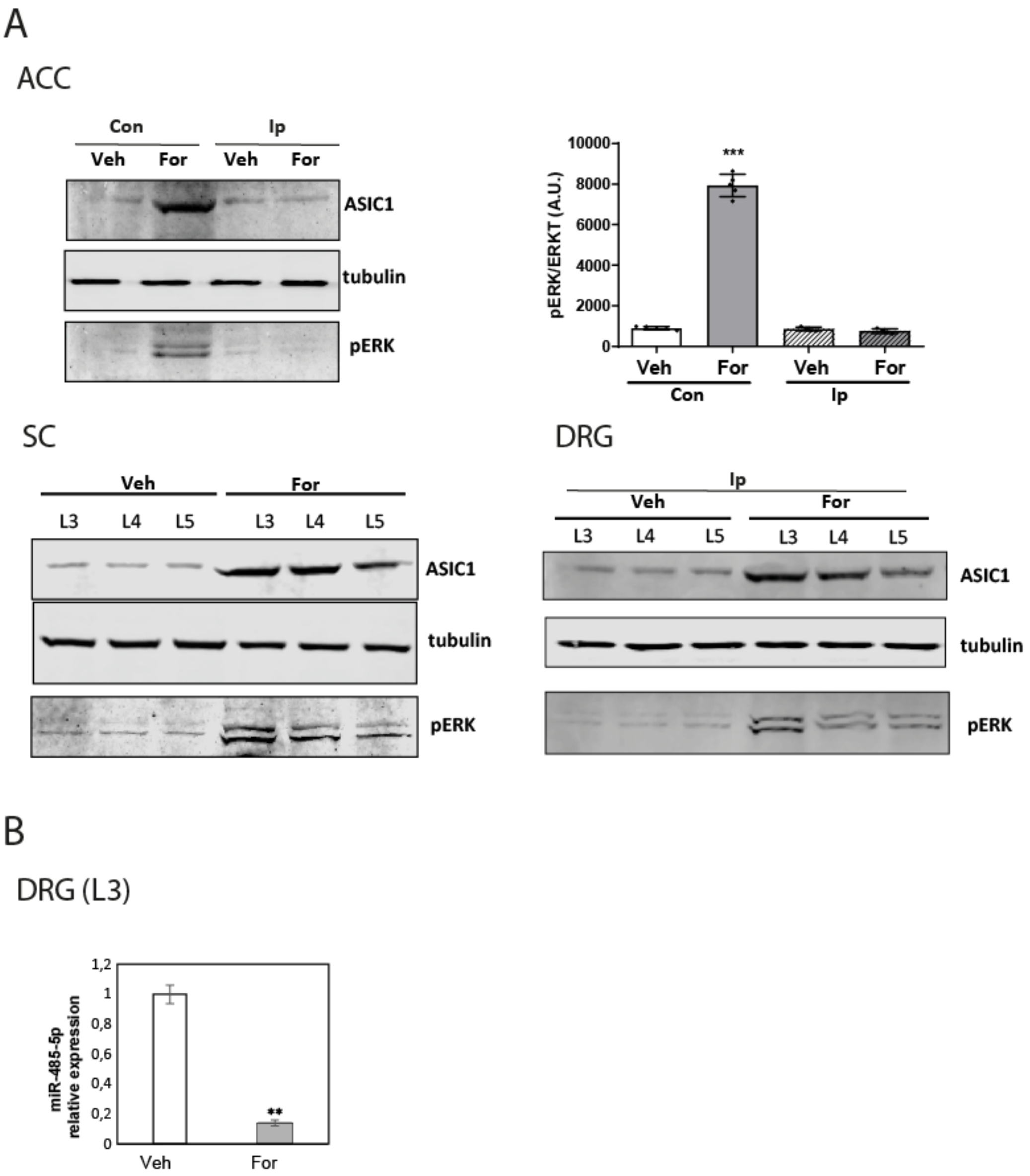

Figure S1 ASIC1 and pERK levels A) Representative membrane of lysates of ACC (top); SC (middle); DRG (bottom) (contralateral (Con or Ip to the injection; and at lumbar levels L3, 4, 5)) tissue from formalin (For) or vehicle (Veh) injected male mice (shown in Figure 2) detected using anti phospho ERK (pERK) antibody, sample loading checked by the tubulin antibody. B) RT-qPCR of miR-485-5p relativized to GAPDH (2ddCT) in DRG at L3, ipsilateral regions to Vehicle (Veh, as control) or formalin injection. Media ± SE of three biological replicates is shown. One from three representative assay (with three biological replicates each) is shown. *** $p < 0.001$, **: $p < 0.01$, two-tailed unpaired t-test

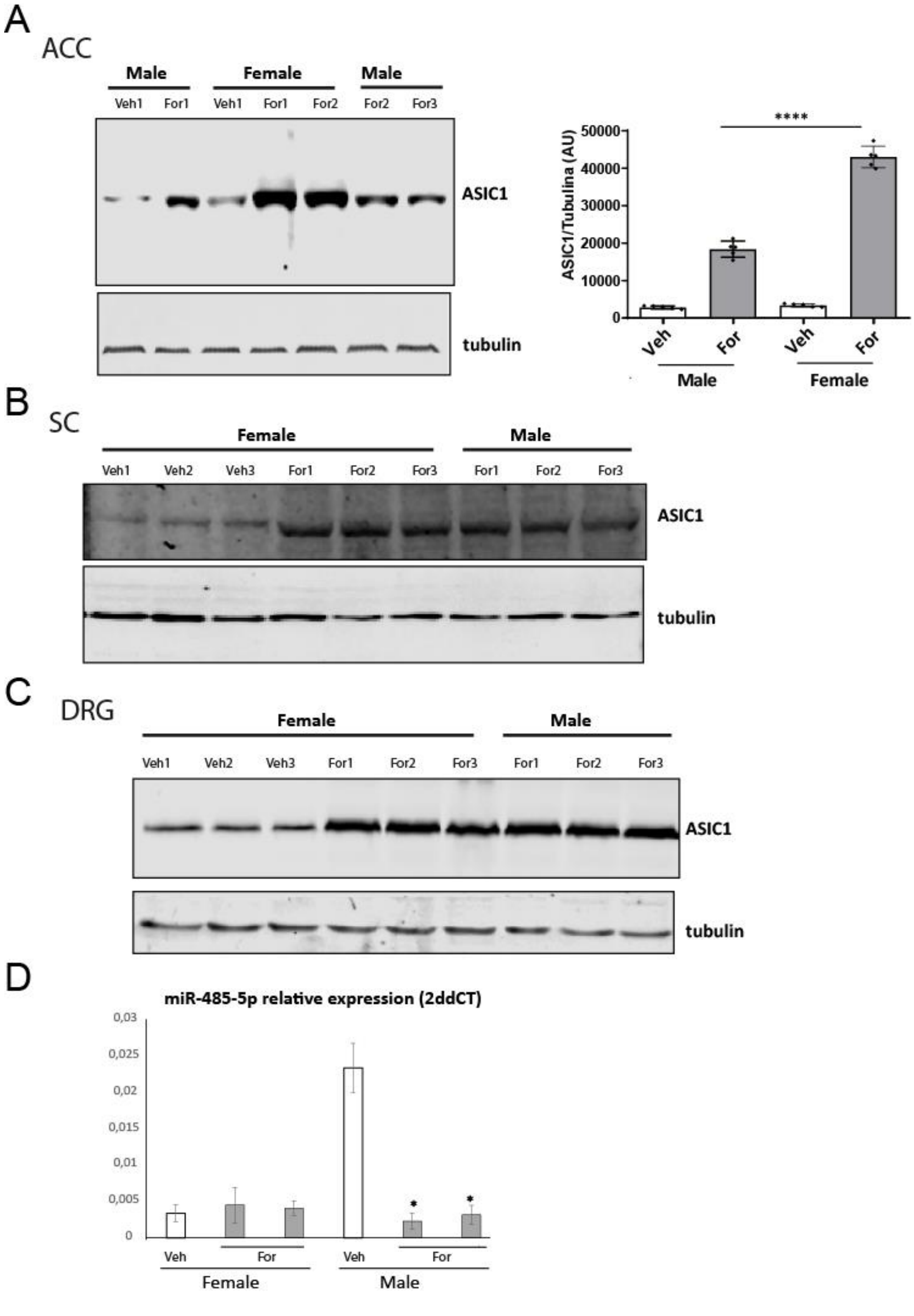

Figure S2 ASIC1 and miR-485-5p levels in male and female mice A) Representative membrane of lysates of ACC (contralateral to the injection) tissue from formalin (For) or vehicle (Veh) injected female and male mice detected using ASIC1 and tubulin antibodies (left), and plot of the results obtained from membranes for ASIC1/tubulin detected levels (right) n=5. B) Representative membrane of lysates of SC and C) DRG (ipsilateral to the injection) from L3 tissue from For or Veh injected female and male mice, no differences were found between male and females in B and C. D) RT-qPCR of miR-485-5p relativized to GAPDH (2ddCT) in ACC contralateral in male and female Veh or For injected mice. Media $\pm$ SE of three technical replicates from two biological replicates of Formalin treated mice for Male and Female are shown. * $p < 0.05$, **** $p < 0.0001$. two-tailed unpaired t-test.
